# Beyond Infection: A Case of Doxycycline-Induced Mania and Prolonged Neuropsychiatric Disturbance

**DOI:** 10.7759/cureus.79671

**Published:** 2025-02-26

**Authors:** Enoch Chi Ngai Lim, Nga Chong Lisa Cheng, Chi Eung Danforn Lim

**Affiliations:** 1 Research and Development, Specialist Medical Services Group, Earlwood, AUS; 2 School of Life Sciences, University of Technology Sydney, Sydney, AUS

**Keywords:** adverse reaction, antibiotics, doxycycline, mania, neuropsychiatric disorders, psychosis

## Abstract

Doxycycline is commonly used in respiratory medicine to treat bacterial lower respiratory tract infections and is also a frequent choice in dermatology for various skin conditions. However, neuropsychiatric adverse reactions to doxycycline are rarely documented in the literature. Even less frequently reported is the persistence of such reactions for up to six weeks after discontinuing a 100 mg doxycycline tablet. We present a rare case of doxycycline-induced mania and psychosis in a patient who had taken the antibiotic for only three days, yet experienced severe neuropsychiatric symptoms that lasted for six weeks after treatment cessation. This case highlights the need for further research to identify the underlying mechanisms, high-risk populations, and implications for informed consent and clinical recommendations.

## Introduction

Doxycycline, a tetracycline antibiotic, is widely used due to its excellent patient tolerability. Although generally well tolerated, like other antibiotics, it carries the potential for adverse effects [1]. However, significant or severe neuropsychiatric reactions to doxycycline are rare. We report a case of a middle-aged Caucasian woman who developed mania and psychosis after taking doxycycline 100 mg daily for three days to treat a lower respiratory tract infection (LRTI).

## Case presentation

A middle-aged Caucasian woman presented for a medical review due to a chronic dry cough and sore throat that had persisted for two months. She had no psychiatric history, active chronic medical conditions, or family history of mental disorders. Based on clinical correlation with physical examination, the clinician diagnosed an LRTI and prescribed a seven-day course of doxycycline at a dose of 100 mg daily.

After three days of taking doxycycline, her husband reported noticeable behavioral changes, including poor sleep patterns, increased energy levels, excessive and continuous talking, and a fixation on certain religious beliefs. Due to her mental and behavioral instability, her family brought her to the local hospital’s emergency department for further evaluation. The initial workup in the emergency room revealed a normal full blood count, CRP, and hepatic and renal function. A chest radiograph showed no radiological evidence of active pneumonia. The psychiatric team established a diagnosis of doxycycline-induced mania by exclusion and advised discontinuation of the medication.

However, six weeks after stopping doxycycline, she continued to experience mood fluctuations. She became tearful easily, exhibited depressive episodes, and had sudden manic episodes characterized by spontaneous singing. Additionally, she engaged in excessive house cleaning and impulsive behaviors, such as an urgent desire to sell her house. Her mental health gradually improved without further treatment, and she fully recovered 10 weeks after doxycycline discontinuation.

## Discussion

This case report highlights that, despite being a widely used broad-spectrum antibiotic with good patient tolerability, doxycycline can cause severe neuropsychiatric adverse reactions. Our case illustrates that doxycycline may induce significant mood disturbances, such as intercalated mania and depressive episodes, even in patients without a prior psychiatric history. In this instance, a potential drug-drug interaction was excluded, as the patient was not taking any other medications or supplements during doxycycline treatment. Furthermore, no underlying chronic medical conditions with pharmacological interaction potential were identified.

Although the exact mechanism remains unclear, suggested explanations include direct neurotoxicity, altered neurotransmitter function, and immune-mediated mechanisms [1]. Tetracyclines possess unique neuroprotective properties, yet studies indicate that doxycycline, due to its lower lipophilicity, carries twice the risk of inducing psychosis compared to minocycline [1]. Notably, the patient’s mood instability persisted for six weeks after doxycycline cessation, suggesting a complex clinical course potentially related to the neuropsychiatric risks associated with less lipophilic tetracyclines [1-3].

Patients prescribed doxycycline should be closely monitored for neuropsychiatric symptoms, with treatment discontinued immediately if such effects emerge. This is particularly important for vulnerable individuals to prevent unwanted neuropsychiatric complications from antibiotic use [2,4]. In cases where symptoms persist after discontinuation, symptomatic supportive management, including psychiatric, pharmacological, and psychological interventions, may be necessary.

As with any medication, clinicians should conduct a thorough risk-benefit assessment before prescribing doxycycline. The patient’s psychiatric and medical history, along with hepatic and renal function, should be considered to ensure appropriate dosing. The necessity of doxycycline should be carefully evaluated, balancing infection severity against the availability of alternative antibiotics with a lower neuropsychiatric risk. Pretreatment counseling is essential to inform patients and caregivers about potential neuropsychiatric reactions and their management. Periodic monitoring is recommended to detect emerging symptoms, with a proactive strategy in place for treatment discontinuation and adjustments if adverse effects occur. Ultimately, the decision to prescribe doxycycline should be based on whether its benefits outweigh the risks, with alternative antibiotics considered when necessary to prioritize both patient safety and effective antimicrobial therapy.

## Conclusions

We report a rare and significant neuropsychiatric adverse reaction to doxycycline in a patient with LRTI, with symptoms persisting for at least six weeks after the last intake of a 100 mg doxycycline tablet. Clinicians should exercise caution and remain vigilant about the potential neuropsychiatric effects of doxycycline, particularly since these reactions may persist long after treatment discontinuation. A thorough risk-benefit assessment should always be conducted when prescribing antibiotics.

This case report contributes to the growing body of evidence on the impact of antibiotics on mental health. It underscores the need for further research to elucidate the underlying mechanisms and identify high-risk groups, ensuring safer clinical practice and informed treatment recommendations.
